# Development of Magnetically Levitated Rotary Table for Repetitive Trajectory Tracking

**DOI:** 10.3390/s22114270

**Published:** 2022-06-03

**Authors:** Fengqiu Xu, Kaiyang Zhang, Xianze Xu

**Affiliations:** Electronic Infromation School, Wuhan University, Wuhan 430070, China; hncxu@whu.edu.cn (F.X.); kyzhang@whu.edu.cn (K.Z.)

**Keywords:** magnetic levitation system, rotary table, disturbance compensation, iterative learning control, trajectory tracking

## Abstract

The magnetic levitation system has been considered as a promising actuator in micromachining areas of study. In order to improve the tracking performance and disturbance rejection of the magnetically levitated rotary table, an iterative learning PID control strategy with disturbance compensation is proposed. The estimated disturbance compensates for the control signals to enhance the active disturbance rejection ability. The iterative learning control is used as a feed-forward unit to further reduce the trajectory tracking error. The convergence and stability of the iterative learning PID with disturbance compensation are analysed. A series of comparative experiments are carried out on the in-house, custom-made, magnetically levitated rotary table, and the experimental results highlight the superiority of the proposed control strategy. The iterative learning PID with disturbance compensation enables the magnetically levitated rotary table to realize good tracking performance with complex external disturbance. The proposed control strategy strengthens the applicability of magnetically levitated systems in the mechanism manufacturing area.

## 1. Introduction

The advantages of no friction, high precision, and cleanliness make magnetic levitation technology attractive in high-precision industrial applications, such as semiconductor lithography, mechanical micromachining, and so on. Nowadays, considering that mechanism manufacturing requires precision translational and rotational motion, different types of magnetically levitated positioning systems have been developed [1,2,3]. The magnetically levitated rotary table (MLRT) is a typical motion control equipment using a magnetically levitated (maglev) actuator, which is suitable to produce the accurate multiaxis motion for micro-machining [4]. However, the magnetic force and torque characteristics are difficult to be described accurately [5,6], and the unmodeled dynamics in the system, such as the non-uniform winding of the coils, the inaccurate magnetizing of the permanent magnet and the measuring noise in the sensing system, inevitably exist in the system design. These adverse factors degrade the motion performance of the maglev positioning system. It is necessary to improve the active disturbance rejection ability for a certain maglev rotary table.

In the past few years, a variety of control strategies have been proposed for the maglev system. Some researchers employ the classical control to design the controllers. Lu et al., employ the PID controller to realize the motion control of the maglev motor [7] and rotary table [4]. Li et al., in [8], use the specified PD control for stabilizing the unstable maglev system and the integral control for eliminating the steady-state error. Kim et al., obtain the full state of the maglev positioning stage via the position sensors, and then employ the state feedback control to realize the precision positioning [9]. Silva-Rivas et al., design a planar maglev system [10], where the Kalman filter is employed for the state estimation and the linear quadratic regulator for the optimal control. Fallaha et al., use the sliding mode control in the maglev system to inherent the measuring noise of the sensors [11]. These controllers are convenient for implementation with acceptable dynamic performance. However, suffering from the model mismatch and external disturbance, the classical control methods cannot realize the satisfactory motion profile. In order to improve the tracking performance of the maglev system, researchers have attempted the advanced control methods. Zhang et al. and Chen et al., propose the adaptive sliding mode controller to deal with uncertainties and improve the robustness for the planar maglev system in [12] and [13], respectively. Basovich et al. in [14], compensate the identified disturbance via an iterative output feedback control strategy to improve the payload capability of the maglev system. Intelligent control algorithms such as neural network and data-driven control can also improve the disturbance rejection ability in a systematic fashion as discussed in [15,16,17]. Even if the various advanced control methods develop continuously, many of them need enough computation resources to promise the real-time solution for the implementation of the controller. Considering the simplicity structure, proportion-integral-derivative (PID) is still the most popular control algorithm in practical engineering with good robustness and high reliability [18]. It is meaningful to study the PID-based MLRT, which improves the practicality of the magnetically levitated technology for industrial application.

In practice, the disturbances and uncertainties resulting from the measuring noise, external contact, varied payload and so on, affect the motion performance of the maglev positioning system for mechanism manufacturing equipment. Obviously, classical PID controller lacks the sufficient disturbance rejection capacity, so the control loop should take some reforms to overcome the shortcomings. It is noted that industrial machines often perform the repetitive trajectory for planar contouring [19,20], such that the iterative learning control (ILC) scheme is an effective feed-forward compensator [21,22,23] for this type of motion task. Additionally, ILC is able to optimize the control signal independent of the accurate system model, which is suitable for the maglev system because the accurate dynamics of the maglev system are hard to model. Therefore, the ILC is employed in the controller to improve the tracking performance for the repetitive trajectory. On the other hand, the disturbance observer has been used successfully for the active disturbance rejection in the industrial applications [16,24], as the influences from the external disturbance and system uncertainties are decreased with the control input signals compensated by the estimated disturbance. Thus, the estimated disturbance is inserted into in the feedback loop of the MLRT controller to inhibit the external disturbance in this work.

An iterative learning PID control strategy with disturbance compensation (LPIDDC) is proposed, which integrates the ILC technique and disturbance compensation (DC) strategy in a series structure. The model-based DC term quickly estimates the disturbances in the system, whereas the data-driven ILC term reduces the impacts of the repetitive uncertainties. This paper focuses on exploring and designing a motion control approach with excellent robustness and tracking ability for the MLRT to meet the requirements of precision industries. The main contributions of the paper are twofold. First, the control strategy which contains a model-based disturbance estimation and a data-driven iterative learning technique is presented to achieve excellent tracking performance. Secondly, the asymptotic stability of the LPIDDC-based maglev system is discussed, and the principle of the determination of control parameters is given.

The rest of the paper is organized as follows. The dynamic model of the maglev rotary table is introduced in Section 2. Section 3 describes the proposed iterative learning PID control method with disturbance compensation. In Section 4, the comparative experiments are carried out to highlight the superiority of the proposed control method. Section 5 gives the conclusions.

## 2. Dynamic Model Description of the MLRT

**Figure 1:**
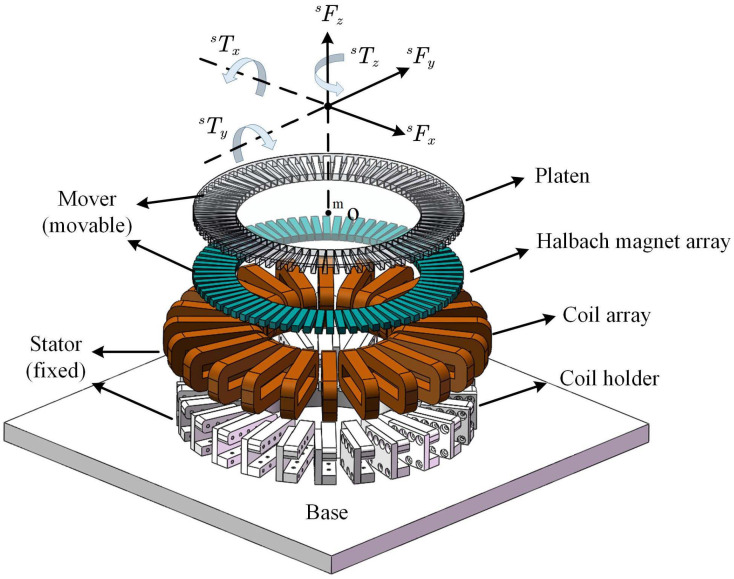
Exploded view of the proposed MLRT.

In this section, the dynamics of the MLRT are analyzed. As shown in Figure 1, the MLRT consists of a circular Halbach permanent magnet (PM) array and 8-phase coils (3 coils for each phase) which can be divided into eight actuator units. Calculation of the magnetic force and torque in an actuator unit is the basis for establishing the magnetic force model of the MLRT. According to the calculation method stated in [25], the magnetic force of an actuator unit is presented in the function related to the position of the table. With the left superscript representing the vector or variable defined in the certain coordinate system, including the magnet coordinate system {m}, the coil coordinate system $\left\{c_{i}\right\}$, and stator coordinate system {s}, the magnetic force is solved by

(1) $${}^{s}f_{i}=-\sum_{q_{c}=0}^{2}{}_{c_{i}}^{s}R\cdot \sum_{g1=1}^{N}\sum_{g2=1}^{N}\sum_{g3=1}^{N}w_{g1}\cdot w_{g2}\cdot w_{g3}\cdot {}^{c_{i}}J\times {}_{m}^{c_{i}}R\cdot {}^{m}B\left({}^{s}p\right),$$

where ${}^{s}f_{i}$ represents the force produced by each actuator unit with the subscript *i* ranging from 1 to 8, $w_{g1}$, $w_{g2}$ and $w_{g3}$ represents the weight of the Gaussian quadrature, *N* is the number of Gauss nodes, ${}^{c_{i}}J$ represents the current density in the i-th phase of coil, ${}^{m}B$ is the magnetic flux density produced by the magnet array in the mover coordinate system depending on the position of the stage ${}^{s}p$, and $q_{c}$ is the index number of the coils. Furthermore, R in Equation (1) represents the rotation transformation matrix between different coordinate systems. Assume the azimuth of the first coil in the i-th coil phase is $\varphi_{i}$ related to the ${}^{s}x\text{-}$axis in the ${}^{s}x^{s}y$ plane, the ${}_{c_{i}}^{s}R$ is solved as

(2) $${}_{c_{i}}^{s}R=\left(\begin{array}{ccc} \cos\left(\varphi_{i}+q_{c}\cdot \frac{\pi }{12}\right) & \sin\left(\varphi_{i}+q_{c}\cdot \frac{\pi }{12}\right) & 0\\ -\sin\left(\varphi_{i}+q_{c}\cdot \frac{\pi }{12}\right) & \cos\left(\varphi_{i}+q_{c}\cdot \frac{\pi }{12}\right) & 0\\ 0 & 0 & 1 \end{array}\right),$$

and ${}_{m}^{c_{i}}R$ is given as,

(3) $${}_{m}^{c_{i}}R{=}_{m}^{s}R\cdot_{s}^{c_{i}}R=\left(\begin{array}{ccc} \cos\left(\gamma_{p}\right) & \sin\left(\gamma_{p}\right) & 0\\ -\sin\left(\gamma_{p}\right) & \cos\left(\gamma_{p}\right) & 0\\ 0 & 0 & 1 \end{array}\right)\cdot_{c_{i}}^{s}R^{-1},$$

where $\gamma_{p}$ is the rotation angle of the stage. Additionally, as the pitch $\alpha_{p}$ and roll $\beta_{p}$ are small enough, the two angles are not considered in the rotation matrices. Then, the resultant force ${}^{s}f_{i}$ can be decomposed into ${}^{s}f_{ix}$, ${}^{s}f_{iy}$, and ${}^{s}f_{iz}$ in the global stator coordinate system. Magnetic torque is the cross product of arm moment ${}^{s}r_{i}$ and magnetic force. It can be written as

(4) $${}^{s}t_{i}={}^{s}r_{i}\times {}^{s}f_{i},$$

where ${}^{s}t_{i}$ represents the resultant torque produced by each actuator unit and ${}^{s}r_{i}$ is the arm moment. The resultant torque contains ${}^{s}t_{ix}$, ${}^{s}t_{iy}$ and ${}^{s}t_{iz}$ in the stator coordinate system.

Now the resultant force and resultant torque of the eight actuator units on each axis can be written as ${}^{s}F_{x}$, ${}^{s}F_{y}$, ${}^{s}F_{z}$, ${}^{s}T_{x}$, ${}^{s}T_{y}$, ${}^{s}T_{z}$ shown in Figure 1.

The dynamics of the MLRT can be regarded as six independent single input, single output (SISO) systems as

(5) $$\left\{\begin{array}{c} {}^{s}F\left(t\right)-{ [0,0,G]}^{T}=m\cdot { [{\ddot{x}}_{p},{\ddot{y}}_{p},{\ddot{z}}_{p}]}^{T}\\ {}^{s}T\left(t\right)=\mathrm{diag}\left(I_{x},I_{y},I_{z}\right)\cdot { [{\ddot{\alpha }}_{p},{\ddot{\beta }}_{p},{\ddot{\gamma }}_{p}]}^{T} \end{array}\right.,$$

where ${}^{s}F$ is ${ [{}^{s}F_{x},{}^{s}F_{y},{}^{s}F_{z}]}^{T}$, ${}^{s}T$ is ${ [{}^{s}T_{x},{}^{s}T_{y},{}^{s}T_{z}]}^{T}$, $[x_{p},\;y_{p},\;z_{p}]$ represents the position of the rotary table, $[\alpha_{p},\;\beta_{p},\;\gamma_{p}]$ means the rotation of the table along each axis, *m* denotes the mass of the mover, *G* is the weight of the mover, and $I_{x}$, $I_{y}$ and $I_{z}$ are the inertia moment of the levitated table related to the corresponding axis. If we obtain the desired force and torque from the control algorithm, the required exciting current in these coils can be calculated via the following formula:

(6) $$I=\Gamma {}^{+}\cdot { [{}^{s}F_{x},{}^{s}F_{y},{}^{s}F_{z},{}^{s}T_{x},{}^{s}T_{y},{}^{s}T_{z}]}^{T}.$$

I is the current vector containing the current in each phase coil. $\Gamma^{+}$ is the pseudoinverse of current-wrench transformation matrix.

After the above analysis, we know that the control object of this article can be described as follows.

(7) $$\theta \cdot \ddot{X}=u+f_{d},$$

where *X* is the position information of the maglev system and represents $x_{p}$, $y_{p}$, $z_{p}$, $\alpha_{p}$, $\beta_{p}$ or $\gamma_{p}$, θ means the normal mass *m* for translational motion or the inertia moment $I_{x}$, $I_{y}$ and $I_{z}$ for rotation, *u* is the control variable which can represents ${}^{s}F_{x}$, ${}^{s}F_{y}$, ${}^{s}F_{z}$, ${}^{s}T_{x}$, ${}^{s}T_{y}$ or ${}^{s}T_{z}$, and $f_{d}$ is the lumped disturbance resulted from the uncertainty and disturbance. Equation (7) can also be written as

(8) $$\left\{\begin{array}{c} {\dot{x}}_{1}=x_{2}=\dot{X}\\ {\dot{x}}_{2}=1/\theta \cdot u+1/\theta \cdot f_{d}=1/\theta \cdot u+D=\ddot{X} \end{array}\right..$$

Rejecting the uncertainties and disturbances in the system and reducing the tracking errors are our concerns.

## 3. LPIDDC Approach

**Figure 2:**
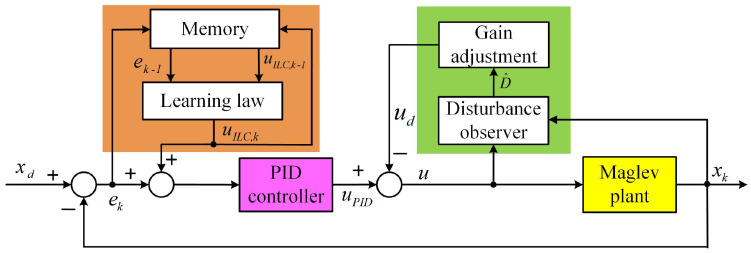
Framework of the proposed LPIDDC control strategy.

To achieve remarkable tracking performance, a hybrid control structure LPIDDC is proposed as shown in Figure 2. The LPIDDC control strategy consists of three parts: PID term, DC term, and ILC term. In the LPIDDC framework, the PID term is responsible for the stability of the maglev system. The DC term is designed based on the plant model, which can effectively estimate and inhibit the disturbance. The ILC term generates the optimal input to change the reference of PIDDC through previous control experience and tracking error, so that the effect of unmodeled repetitive disturbance is reduced. Compared with the conventional feed-forward–feedback control, for example LPID, the DC term not only enhances the disturbance rejection capability, but also eliminates the system errors which cannot be totally removed by the ILC. The LPIDDC control framework offers an effective motion control technology scheme for the industrial application of the maglev system and also supplies a meaningful idea for control engineers.

In this framework, if the ILC term or the DC term is paused, the LPIDDC control strategy becomes the PID with disturbance compensation (PIDDC) or the iterative learning feed-forward PID (LPID) control scheme. If both of the ILC term and the DC term are switched off, only the PID feedback controller works. This flexible control framework is helpful for us to carry out the comparative experiments.

### 3.1. PID Term

When the dynamic decoupling unit works, the MLRT is equivalent to six independent SISO second-order systems. Considering the second-order system is open-loop unstable, a feedback compensator is necessary to stabilize the unstable maglev system. The classical PID controller is employed because of the simple structure and reliable performance, meanwhile the PID method is also robust for the measuring noise and unmodeled dynamics. In order to obtain the desired response, we solve the control parameters via a tool named PID TUNER provided by MATLAB. This tool automatically calculates the control parameters based on the required motion performance in the time domain. The designer describes the time-domain performance by two parameters: response time and transient behavior. The response time can be set directly, and a short value requires a large open-loop gain but may result in the input saturation. The transient behavior is given by a ratio to rate the weights of robustness and aggressiveness. The obtained transfer function is defined in the *s* domain, and it will be converted into the discrete domain via the zero-order-hold with the certain sampling frequency. Then, the transfer function can be implemented in the controller, which calculates the control signal at each sampling cycle with the positions and rotational angles from the sensing system being the inputs. The obtained control signal, the $u_{\mathrm{PID}}$ in Figure 2, represents the required force or torque for the motion control, and it will be employed to solve the required excitation current for each coil phase via the decoupling unit by Equation (6).

### 3.2. Disturbance Compensation Term

There are many uncertain factors, such as unmodeled nonlinear dynamics and external disturbances in the actual industrial environment. A nonlinear disturbance observer is designed to estimate these disturbances and provide the feed-forward compensation. The nonlinear disturbance observer can be expressed as

(9) $$\left\{\begin{array}{c} \hat{D}=r_{0}+p\left(x_{1},x_{2}\right)\\ {\dot{r}}_{0}=-L\left(x_{1},x_{2}\right)\cdot r_{0}-L\left(x_{1},x_{2}\right)\cdot [p\left(x_{1},x_{2}\right)+\frac{1}{\theta }\cdot u] \end{array}\right.,$$

where $\hat{D}$ is the estimated disturbance, $L\left(x_{1},x_{2}\right)$ is the gain of disturbance observer, and $p\left(x_{1},x_{2}\right)$ is a nonlinear function. Their relationship is given as

(10) $$L\left(x_{1},x_{2}\right){\dot{x}}_{2}=\dot{p}\left(x_{1},x_{2}\right).$$

In general, the actual disturbance *D* can be considered, which varies slowly at the steady state,

(11) $$\dot{D}\approx 0.$$

Thus, the observation error is

(12) $$e_{d}=D-\hat{D}.$$

Substituting Equations (9)–(11) into Equation (12), we can get the dynamic equation of observation error,

(13) $$\begin{array}{cc} {\dot{e}}_{d} & =\dot{D}-\dot{\hat{D}}=-{\dot{r}}_{0}-\dot{p}\left(x_{1},x_{2}\right)\\ \; & =L\left(x_{1},x_{2}\right)\cdot [r_{0}+p\left(x_{1},x_{2}\right)]-L\left(x_{1},x_{2}\right)\cdot \left({\dot{x}}_{2}-\frac{u}{\theta }\right)\\ \; & =-L\left(x_{1},x_{2}\right)\cdot e_{d}\;. \end{array}$$

By solving Equation (13), we get

(14) $$e_{d}=e_{d}\left(0\right)\cdot e^{-L\left(x_{1},x_{2}\right)\cdot t},$$

where $e_{d}\left(0\right)$ is the observation error at the beginning instant of each repetitive trajectory. If $L\left(x_{1},x_{2}\right)=\sigma \left(\sigma >0\right)$, the observed error converges exponentially. Then, $p\left(x_{1},x_{2}\right)$ is given as

(15) $$p\left(x_{1},x_{2}\right)=\sigma \cdot x_{2}.$$

The output of the observer should be transmitted to the gain adjustment module so that the observed disturbance can be converted into corresponding control variables,

(16) $$u_{d}=\hat{D}\cdot \theta .$$

After implementing the disturbance observer, the net control input of the system is $u_{\mathrm{PID}}-u_{d}$ as illustrated in Figure 2. Thus, the second equation in Equation (8) can be expressed as

(17) $${\dot{x}}_{2}=\frac{1}{\theta }\left(u_{\mathrm{PID}}-u_{d}\right)+D=\frac{1}{\theta }u_{\mathrm{PID}}+e_{d}=\frac{1}{\theta }\left(u_{\mathrm{PID}}+d\right),$$

where *d* is the equivalent residual disturbance, which is considered to be acting on the control input signal and approaching to 0 at the steady state. It can be seen from Equation (17) that the disturbance in the system is changed from *D* to $e_{d}$ when the disturbance observer works. In practice, if a lot of high-frequency noise exists in the system, the signal observed by the disturbance observer is required to be processed by a low-pass filter to obtain a more accurate estimated value.

### 3.3. Iterative Learning Term

When the MLRT undertakes the repetitive trajectory tracking, the tracking performance can be improved significantly by learning the tracking errors from the previous iterations in the framework of ILC. ILC is a model-free feedforward control method, and it directly compensates the control input with the existing feedback controller not modified. The iterative learning control is expressed as

(18) $$u_{\mathrm{ILC},k}=Q\cdot \left(u_{\mathrm{ILC},k-1}+G_{\mathrm{ILC}}\cdot e_{k-1}\right),$$

where $u_{\mathrm{ILC},k}$ is the ILC signal at the k-th repetitive tracking, *Q* is a low-pass filter which can suppress the system noise, and $G_{\mathrm{ILC}}$ is a *s*-domain transfer function which processes the tracking error to realize the learning function. The following Theorem presents the convergence of tracking errors with the MLRT regulated by the compound control method containing the PID, disturbance compensation, and iterative learning control.

**Theorem** **1.** * Considering the MLRT described by Equation (7), the proposed control strategy which consists of the PID term, the DC term and the ILC term can guarantee the convergence of the tracking error.*

**Proof of Theorem** **1.** Noting the control block diagram in Figure 2 and the dynamics in Equation (17), the dynamic of the maglev system can be described as

(19) $$x_{k}=N_{r}\cdot x_{d}+N_{r}\cdot u_{\mathrm{ILC},k}+N_{u}\cdot d,$$

where $x_{k}$, $x_{d}$ are output and reference, respectively. The transfer functions $N_{r}$ and $N_{u}$ are expressed as

(20) $$N_{r}=\frac{G(s)\cdot C(s)}{1+G(s)\cdot C(s)\prime }$$

(21) $$N_{u}=\frac{G(s)}{1+G(s)\cdot C(s)\prime }$$

where G(s) and C(s) represent transfer function of the maglev system and the PID controller, respectively. The tracking error $e_{k}$ is equal to $x_{d}-x_{k}$, and substituting Equation (18) into Equation (19), then $e_{k}$ is written as

(22) $$e_{k}=x_{d}-x_{k}=x_{d}-N_{r}\cdot x_{d}-Q\cdot N_{r}\cdot u_{\mathrm{ILC},k-1}-Q\cdot N_{r}\cdot G_{\mathrm{ILC}}\cdot e_{k-1}-N_{u}\cdot d.$$

Replacing the subscribe *k* in Equation (19) by k−1, the following formula is obtained.

(23) $$-N_{r}\cdot u_{\mathrm{ILC},k-1}=e_{k-1}+\left(N_{r}-1\right)\cdot x_{d}+N_{u}\cdot d.$$

Substituting the term, $-N_{r}\cdot u_{\mathrm{ILC},k-1}$, of Equation (23) into the right side of Equation (22), the following equation is derived:

(24) $$e_{k}=Q\cdot \left(1-N_{r}\cdot G_{\mathrm{ILC}}\right)\cdot e_{k-1}+\left(1-N_{r}\right)\cdot \left(1-Q\right)\cdot x_{d}+\left(Q-1\right)\cdot N_{u}\cdot d.$$

The $x_{d}$ is the constant reference, and the residual disturbance *d* can be 0 with an appropriate disturbance observation gain. Therefore, we have

(25) $$e_{k+1}-e_{k}=Q\cdot \left(1-N_{r}\cdot G_{\mathrm{ILC}}\right)\cdot \left(e_{k}-e_{k-1}\right).$$

With the reasonable transfer functions *Q* and $G_{\mathrm{ILC}}$, ${∥Q\cdot \left(1-N_{r}\cdot G_{\mathrm{ILC}}\right)∥}_{\infty }<1$. In this case, the system error approaches to 0 with the iteration times increasing. □

In this work, a general PD-type tunable ILC learning function is employed. The ${∥Q\cdot \left(1-N_{r}\cdot G_{\mathrm{ILC}}\right)∥}_{\infty }$ can be less than 1 by tuning the control parameter, which promises the convergence of the tracking errors. Furthermore, the experimental results highlight that the obtained controller makes the tracking errors of the repetitive reference converge into a bounded range.

### 3.4. Summary of LPIDDC Control Strategy

The control strategy proposed in this paper can be regarded as a feed-forward–feedback composite controller. The controller includes a PID feedback term with disturbance compensation and iterative learning feedforward term. The feedforward and feedback terms can complement each other and work together. For the industrial fields which have adopted the PID controller, we only need to add DC and ILC terms into the controller without redesigning the existing equipment.

The feedback controller is responsible for the stabilization of the system, whereas the disturbance compensation realizes the active disturbance rejection and the ILC unit improves the tracking performance for the repetitive motion tasks. Therefore, the MLRT can carry out the the precision repetitive tracking task when the system suffers from the external complex disturbance.

## 4. Experimental Studies

### 4.1. Hardware Setup

**Figure 3:**
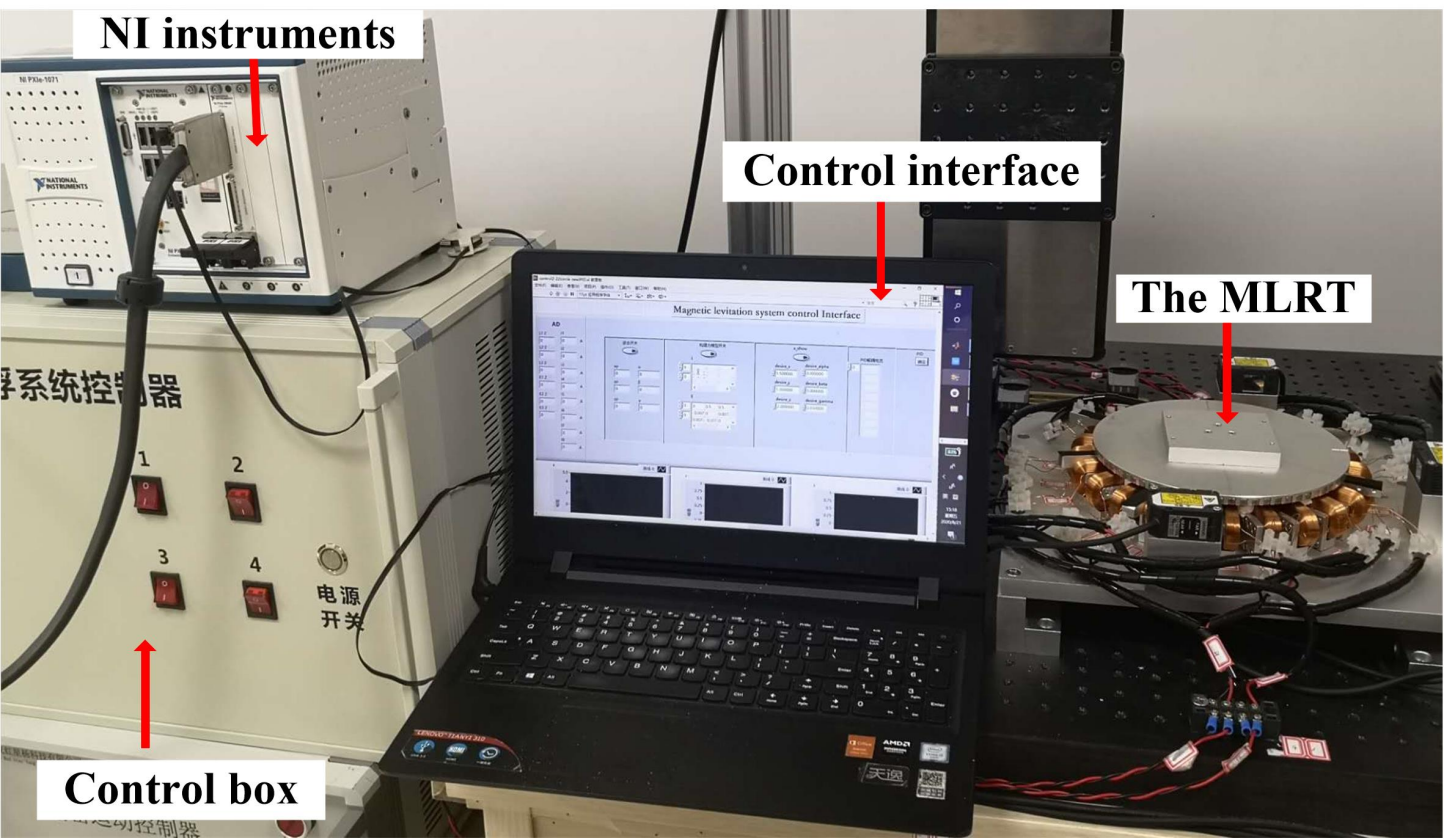
Experimental setup.

In order to test the performance of the proposed control algorithm, including disturbance rejection and trajectory tracking performance, a series of comparative experiments are carried out on the MLRT given in Figure 3. Six laser-displacement sensors are employed to obtain the position and rotation informations of the rotary table. To reduce the impact of measuring noise on the system, the median filter algorithm processes the raw data from the sensors. The currents exciting the coils are provided by eight independent power amplifiers with current limitation of 4 A. The control strategies for the MLRT are implemented on the data processing platform NI PXIe-8880 and a reconfigurable I/O module NI PXIe-7856R, which are manufactured by National Instrument. Six channels of PXIe-7856R are configured as analog input to collect the sensors’ signals and the other eight channels are configured as analog output to drive these power amplifiers. The NI PXIe-8880 realizes the certain control algorithm when it receives the operation commands from the PC. Additionally, the sampling frequency of the control algorithms running on NI PXIe-8880 is 1 kHz.

To verify the effectiveness of the proposed control strategy, the PID controller, PIDDC controller, LPID controller and the proposed LPIDDC controller regulate the MLRT to undertake the same trajectory tracking tasks.

M1: PID: With the response time given as 15ms and the ratio between the robustness and aggressiveness set as 9:1, the PID parameters can be solved automatically. Utilizing the obtained control parameters, the system has a phase margin of $68.5^{\circ }$ at a crossover frequency of 21Hz.

M2: PIDDC: Based on the PID method, this controller employs the DC terms to compensate the control input signal. The DC term enhances the disturbance rejection capability. In the DC term, the value of observation gain σ should be determined carefully. A larger σ amplifies the noise in the system, whereas a small one decreases the estimation accuracy. In this paper, σ is chosen as 1000.

M3: LPID: Based on the PID method, the ILC term is employed as the feed-forward unit. The Q-filter in the ILC term is set as the typical second-order low-pass filter whose cut-off frequency is 60Hz and damping ratio is 0.707. In the learning function $G_{\mathrm{ILC}}$, $k_{\mathrm{ip}}=0.05$ and $k_{\mathrm{id}}=0.001$.

M4: LPIDDC: The proposed controller is presented in Section 2. For a fair comparison, the control parameters of the PID term, DC term, and ILC term are the same in the four controllers.

The MLRT is equivalent to six independent SISO systems, and the performance of translation and rotation on each axis are similar. In the following test, we choose the vertical motion and rotation around the vertical axis to present the performance of different control methods.

(1) Track1: the MLRT is controlled to track the sinusoidal trajectory in ${}^{s}z$-axis below with the unit being mm,
  (26) $$z_{d}=3+0.5\sin\left(\pi t\right),$$
  which has an an angular speed of ω=3.14rad/s and a velocity of $v=1.57\cos\left(\pi t\right)\;\mathrm{mm}/s$.
(2) Track2: the MLRT is controlled to track the sinusoidal trajectory in ${}^{s}\gamma$-axis below with unit being rad,
  (27) $$\gamma_{d}=0.1\sin\left(\pi t\right),$$
  which has an angular speed of ω=3.14rad/s and a velocity of $v=0.314\cos\left(\pi t\right)\mathrm{rad}/s$.

For the following quantitative analysis, the related indexes are employed.

(1) $e_{\mathrm{RMS}}=\sqrt{\frac{1}{T}\int_{0}^{T}{\left|x_{d}\left(t\right)-x_{k}\left(t\right)\right|}^{2}dt}$, the root-mean-square value of the trajectory tracking error, where *T* is the period of tracking trajectory.
(2) $e_{M}=\max\left\{\left|x_{d}\left(t\right)-x_{k}\left(t\right)\right|\right\}$, the maximal absolute value of the trajectory tracking error.

It is noted that all performance indices are obtained by calculating the trajectory tracking error of the last cycle, which is from the time instant 22s to 24s.

### 4.2. Trajectory Tracking without External Disturbance

To test the feasibility of these control strategies, the MLRT is controlled to track the trajectory without external disturbance. Because the mover of the rotary table is suspended, the mechanical friction does not exist. The MLRT can be regarded as working in an ideal environment.

**Figure 4:**
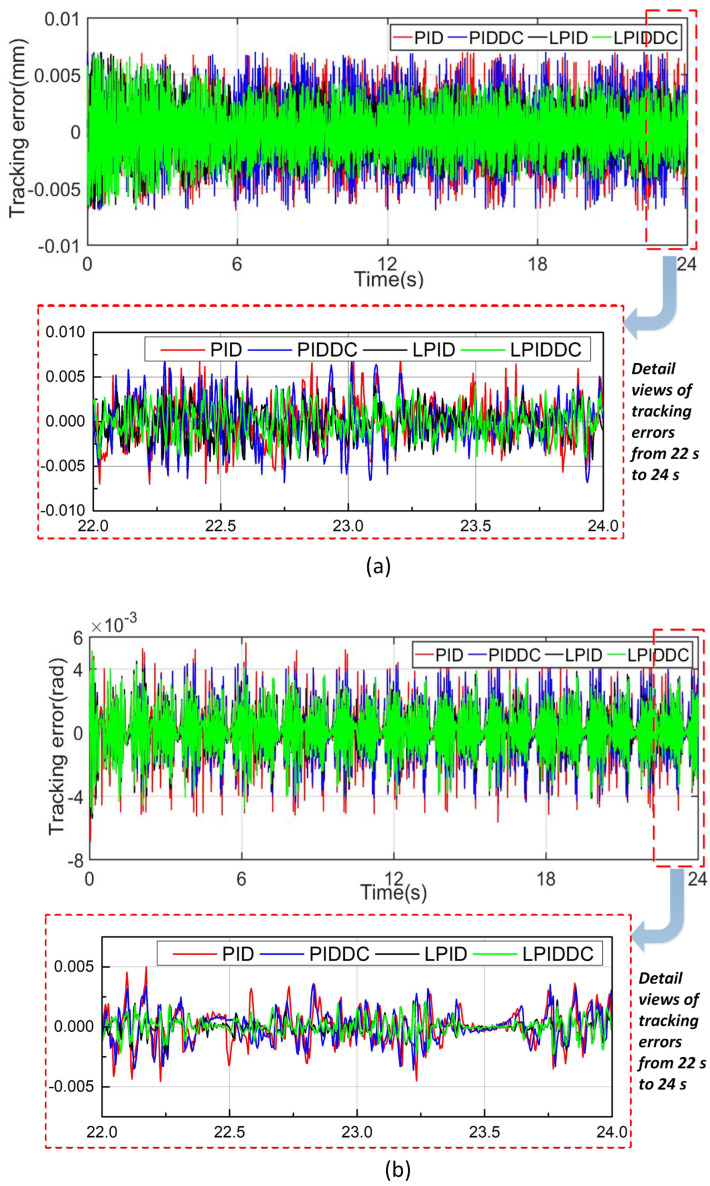
Tracking errors of the MLRT without external disturbance. (**a**) Tracking error of Track1. (**b**) Tracking error of Track2.

The trajectory tracking errors of Track1 and Track2 are plotted in Figure 4a and Figure 4b respectively. The motion indices are listed in Table 1. As seen in Figure 4, we conclude that all control strategies are effectively implemented. In this case, the $e_{\mathrm{RMS}}$ of M1 and M2 are almost same due to the fact that the DC term does not play a significant role when there is no external disturbance. M3 and M4 perform better than M1 benefiting from the feed-forward compensation of the ILC term. In Track1, $e_{\mathrm{RMS}}$ of M3 and M4 decrease 37.82% and 39.15% referring to M1, whereas $e_{M}$ of M3 and M4 are 63.61% and 61.92% of M1. In Track2 $e_{\mathrm{RMS}}$ of M3 and M4 decrease 34.60% and 34.74% referring to M1, while $e_{M}$ of M3 and M4 are 45.71% and 43.83% of M1 respectively. In summary, all control strategies are feasible for the maglev rotary table, but the proposed LPIDDC control performs better without the external disturbance.

### 4.3. Trajectory Tracking with External Disturbance

#### 4.3.1. Step Disturbance

In industrial applications, the maglev rotary table may suffer from the sudden disturbance because of some unexpected events, which increases the tracking error of the MLRT. In order to simulate this situation, the external force and torque disturbances are added to the obtained control signal, the *u* in Figure 2, before it enters into the maglev system. Then, the four different control methods can be evaluated by comparing the different tracking performances. When the MLRT undertakes Track1, a force of 1.5N is added to the obtained control signal on *z*-axis at 5-th second and removed at 6-th seconds. Similarly, to test the robustness of the controllers in ${}^{s}\gamma$-axis, a torque of 1450N·mm is added and removed at the same time in the ${}^{s}\gamma$ direction when the MLRT undertakes Track2.

**Figure 5:**
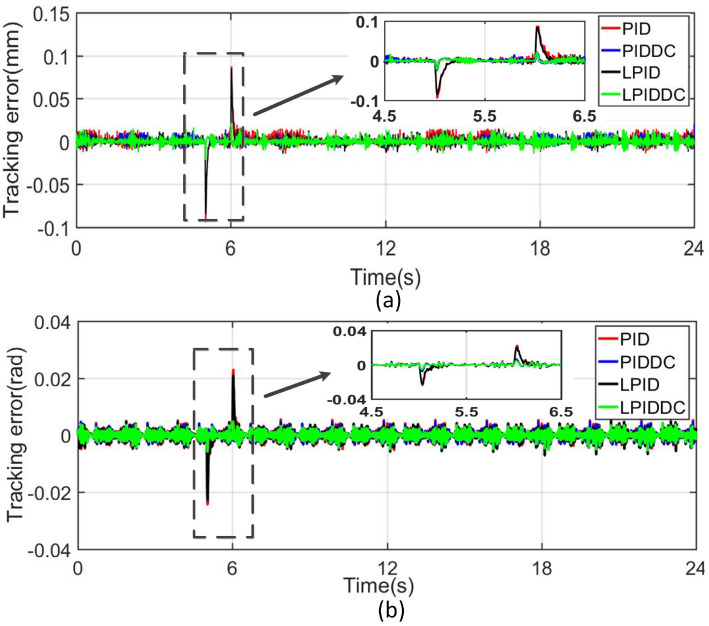
Tracking results of Track1 and Track2 with step disturbance. (**a**) Tracking error in ${}^{s}z$-axis. (**b**) Tracking error in ${}^{s}\gamma$-axis.

The tracking errors are shown in Figure 5. These figures obviously demonstrate that M1 and M3 cannot suppress the sudden disturbance. The peak values of M1 and M3 are about 85μm in Track1 and 22mrad in Track2. With the DC term, M2 and M4 are robust to the disturbance as the peak error of M2 and M4 are about 21μm in Track1 and 6mrad in Track2. It is concluded that the DC term in the proposed control strategy can effectively deal with this step disturbance.

#### 4.3.2. Complex Disturbance

To further test the disturbance rejection capability of the proposed control strategy, the complex external disturbances given below are imposed on the maglev system in the form of force or torque.

(28) $$F_{\mathrm{dis}}=\sin\left(2z_{p}\right)+\sin\left(4\pi t\right)+\mathrm{arccot}\left(z_{p}\right)+e^{-z_{p}}\left(N\right),$$

(29) $$T_{\mathrm{dis}}= [\sin\left(2\pi t+\pi /2\right)+\cos\left(3\gamma_{p}+\pi /2\right)+e^{-10\gamma_{p}}\left.+\arctan\left(20\gamma_{p}\right)+30\gamma_{p}^{2}+\sin\left(\pi t\right)+1.5\right]\times 600\left(N\cdot \mathrm{mm}\right),$$

where $F_{\mathrm{dis}}$ is the disturbance in ${}^{s}z$ direction when the MLRT moves along Track1, and $T_{\mathrm{dis}}$ is the disturbance in ${}^{s}\gamma$ direction when the MLRT rotates along Track2. These disturbances can be regarded as the nonlinear function related to the system state and time.

**Figure 6:**
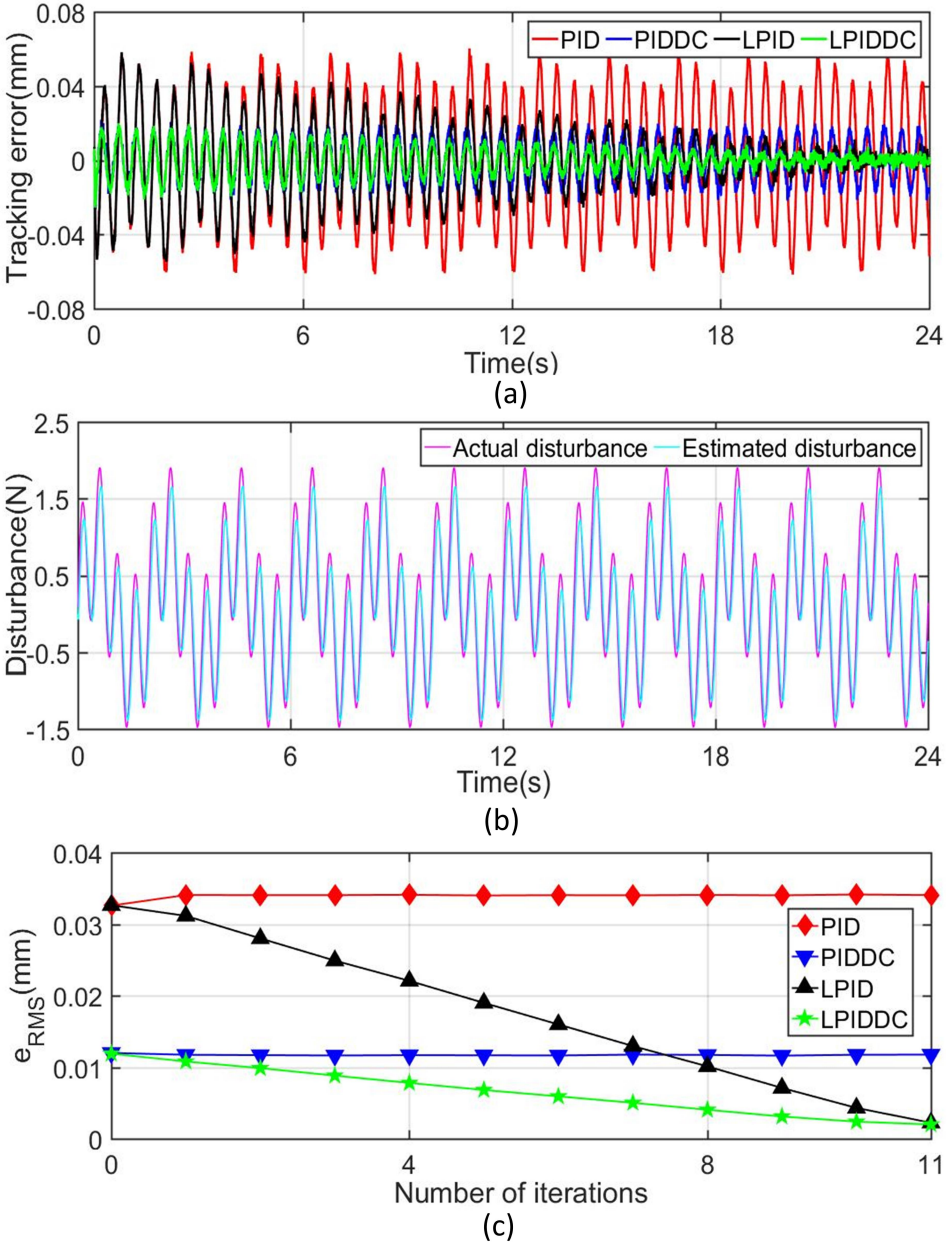
Tracking results of Track1 with complex disturbance. (**a**) Tracking error in ${}^{s}z$-axis. (**b**) Disturbance quantity and its estimated value. (**c**) Convergence rate of $e_{\mathrm{RMS}}$ in ${}^{s}z$-axis.

**Figure 7:**
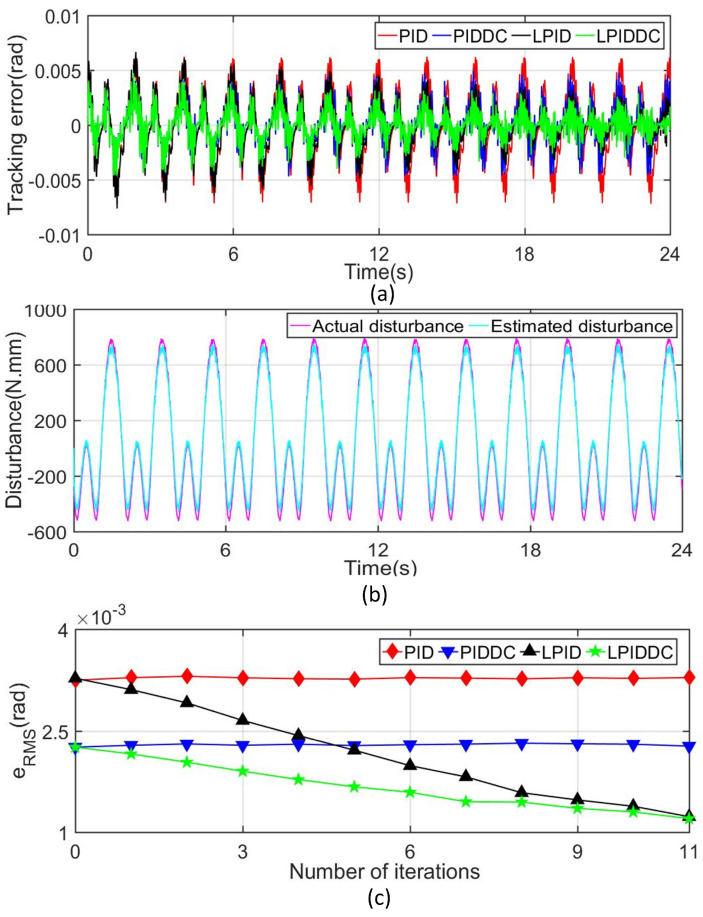
Tracking results of Track2 with complex disturbance. (**a**) Tracking error in ${}^{s}\gamma$-axis. (**b**) Disturbance quantity and its estimated value. (**c**) Convergence rate of $e_{\mathrm{RMS}}$ in ${}^{s}\gamma$-axis.

The tracking errors of the MLRT in Track1 and Track2 are shown in Figure 6a and Figure 7a respectively. In order to facilitate the quantitative analysis, the related tracking indices under different controllers at the 11th iteration are also listed in Table 2. These results show that the classical PID controller can not deal with the complex external disturbance. Figure 6b and Figure 7b present that the disturbance observer works well so that the active disturbance rejection of the MLRT is improved. Although the final tracking error of the LPID controller decreases after several iterations, its initial tracking error does not meet the requirements as illustrated in Figure 6c and Figure 7c. Furthermore, if the complex disturbances are not periodic, the LPID controller cannot reduce the tracking error of the system via the ILC term. Benefiting from the DC term, LPIDDC can overcome the shortcomings of LPID. In Track1, $e_{\mathrm{RMS}}$ of M4 is 2.001μm, which decrease 82.98% and 10.59% referring to M2 and M3. In Track2, $e_{\mathrm{RMS}}$ of M4 is 1.587mrad, which decreases 22.01% and 12.75% referring to M2 and M3. In Track1 and Track2, $e_{M}$ of M4 are smallest compared with that of other controllers. The above results highlight that the proposed control strategy improves the tracking performance when the MLRT suffers from complex external disturbances, and this improvement make the maglev more suitable for the precision manufacture area.

#### 4.3.3. Disturbance Caused by Polyfoam

**Figure 8:**
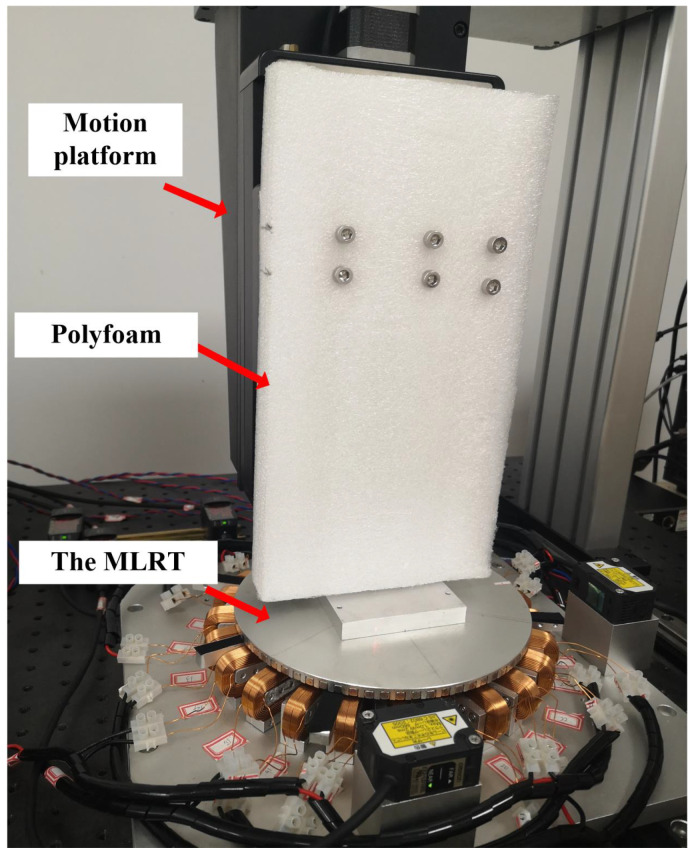
Photo of the MLRT with polyfoam producing unknown disturbance. Experimental video is found in the Supplementary Video S1.

In order to simulate the unknown disturbances in the industrial field, a polyfoam is fixed above the magnetic suspension rotary table as shown in Figure 8. When the MLRT tracks the sinusoidal trajectory in the ${}^{s}z$-direction like Track1, the polyfoam produces the random force acting on the rotary table, which is considered as the unknown disturbance. This test can investigate the performance of the proposed controller for this unknown disturbance.

**Figure 9:**
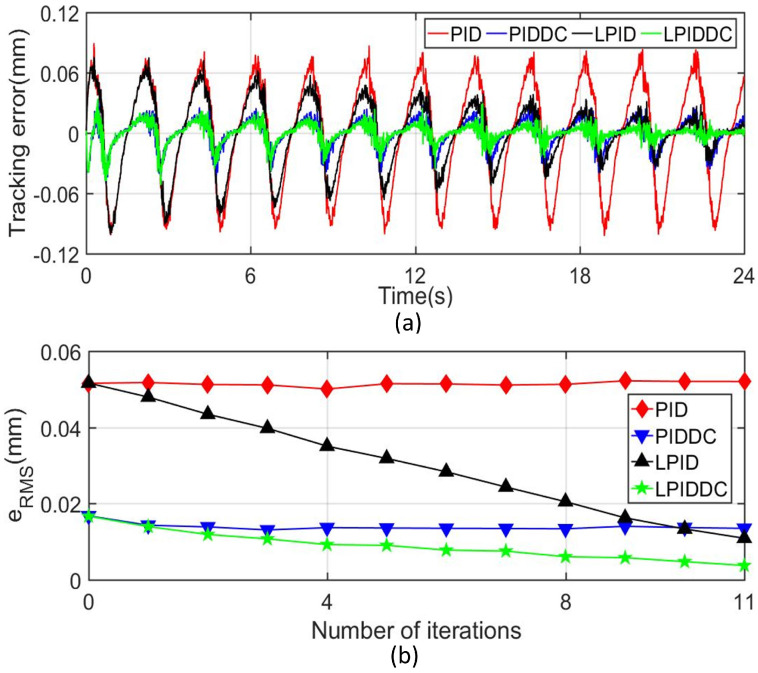
Tracking results of Track1 with polyfoam disturbance. (**a**) Tracking error in ${}^{s}z$-axis. (**b**) Convergence rate of $e_{\mathrm{RMS}}$ in ${}^{s}z$-axis.

The tracking errors plotted in Figure 9a illustrate that M4 has a smaller tracking error compared with the other three controllers. Meanwhile, the tracking indexes of the different controller given in Table 3 demonstrate that the ($e_{\mathrm{RMS}}$ and $e_{M}$) of M2 and M3 are smaller than M1, and M4 outperforms the other three controllers. The $e_{\mathrm{RMS}}$ of M4 is 92.80% smaller than M1, 72.22% smaller than M2, and 65.59% smaller than M3. Figure 9b also shows that LPID and LPIDDC controller reduce the error with the iteration number increasing, and the introduction of the DC term accelerates the convergence speed of the system error. We conclude that these results demonstrate that the proposed LPIDDC controller has excellent disturbance rejection capability for unknown external disturbances.

#### 4.3.4. Circle Trajectory Tracking

**Figure 10:**
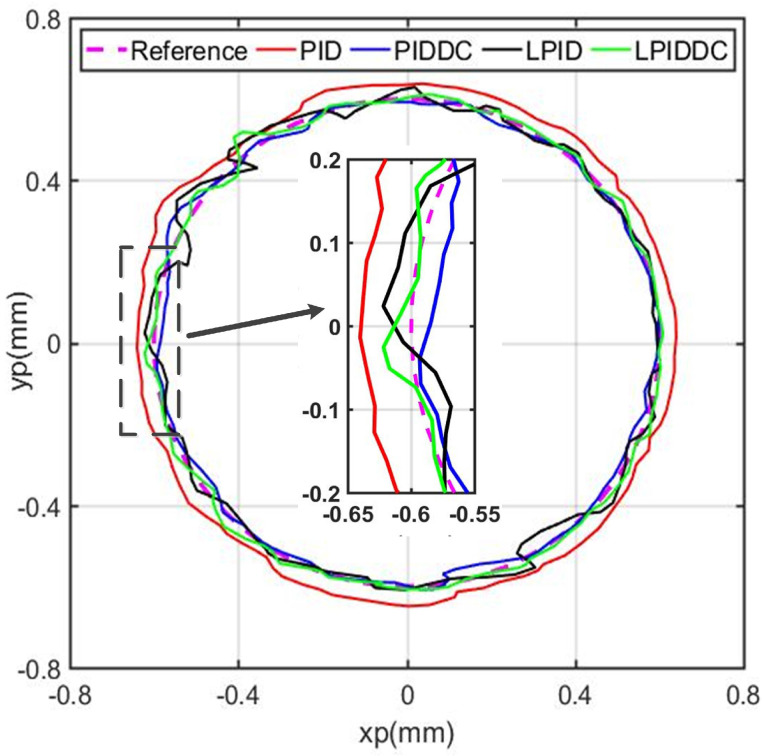
Circular trajectory tracking of the MLRT.

In this case study, the MLRT is controlled to track a circular trajectory in the horizontal plane shown in Figure 10. The circular contour is formed by making the MLRT track a cosine trajectory in ${}^{s}x$-axis and a sinusoidal trajectory in ${}^{s}y$-axis simultaneously. The trajectory is given as:

(30) $$\left\{\begin{array}{c} x_{d}=0.6\cdot \cos\left(5\pi t\right)\;\;\left(\mathrm{mm}\right)\\ y_{d}=0.6\cdot \sin\left(5\pi t\right)\;\;\left(\mathrm{mm}\right) \end{array}\right..$$

The radius of the circular profile is 0.6mm and the angular velocity is 15.71rad/s. The tracking error is the shortest distance from the actual position to the desired position in the contour. The MLRT undertakes the repetitive circular trajectory, and Figure 10 and Table 4 are the tracking performances and working indexes of the rotary table the in the period of 4.4 s∼4.8s from the start time point.

The tracking error of M1 is large due to the fact that the PID controller cannot reject the motion coupling of the multiple axes tracking effectively. Although M2, M3, and M4 all achieve smaller tracking errors than M1, M4 still has the best trajectory tracking performance as the $e_{\mathrm{RMS}}$ of M4 is 40.38%, 57.30%, and 68.23% of M1, M2, and M3, respectively, wheresa that of $e_{M}$ is 64.40%, 75.02%, and 65.78%. From the partial view in Figure 10, the LPIDDC contour is more closed to the desired circle than others. Based on the above experimental results, we can conclude that the proposed control strategy is effective for the planar motion of the magnetic levitation rotary table.

## 5. Conclusions

In order to develop the practicality of MLRT, an iterative learning PID control method with disturbance compensation is proposed. The proposed LPIDDC consists of a PID term, a DC term, and an ILC term. The PID term guarantees the stability of the maglev system. The DC term estimates the disturbance in the system and corrects the control signal to suppress the impact of disturbance. The ILC term compensates for the unknown repetitive disturbances and uncertainties in the system to improve the repetitive trajectory tracking performance. Comparative experiments between four controllers are carried out on the MLRT, and these experimental results indicate that the proposed control algorithm improve the tracking performance for the repetitive trajectory with complex disturbance, which promotes the application of magnetically levitated technology in the area of micro milling, microgrinding, etc.

## Figures and Tables

**Table 1:** Tracking performance of the MLRT without external disturbance.

Trajectory	Track1	Track1	Track2	Track2
Index	$e_{\mathrm{RMS}}\;\left(\mu m\right)$	$e_{M}\;\left(\mu m\right)$	$e_{\mathrm{RMS}}\;\left(\mathrm{mrad}\right)$	$e_{M}\;\left(\mathrm{mrad}\right)$
M1	3.083	6.996	2.159	4.999
M2	3.076	6.984	2.161	3.611
M3	1.917	4.450	1.412	2.285
M4	1.876	4.332	1.409	2.191

**Table 2:** Tracking performance of the MLRT with complex disturbance.

Trajectory	Track1	Track1	Track2	Track2
Index	$e_{\mathrm{RMS}}\;\left(\mu m\right)$	$e_{M}\;\left(\mu m\right)$	$e_{\mathrm{RMS}}\;\left(\mathrm{mrad}\right)$	$e_{M}\;\left(\mathrm{mrad}\right)$
M1	34.058	59.915	2.918	5.418
M2	11.759	21.527	2.035	2.883
M3	2.238	9.373	1.819	2.379
M4	2.001	7.445	1.587	1.907

**Table 3:** Tracking performance of the MLRT with polyfoam disturbance for Track1.

Index	$e_{\mathrm{RMS}}\left(\mu m\right)$	$e_{M}\left(\mu m\right)$
M1	52.034	97.302
M2	13.496	36.248
M3	10.896	31.637
M4	3.749	17.547

**Table 4:** Tracking performance of the MLRT for circular trajectory.

Index	$e_{\mathrm{RMS}}\;\left(\mu m\right)$	$e_{M}\;\left(\mu m\right)$
M1	59.869	96.935
M2	35.142	83.218
M3	29.511	94.900
M4	20.135	62.428

## Data Availability

Not applicable.

## Supplementary Materials

The following supporting information can be downloaded at: https://www.mdpi.com/article/10.3390/s22114270/s1, Video S1 is a supporting video for Figure 8.

## Abbreviations

The following abbreviations are used in this manuscript:

MLRT	Magnetically levitated rotary table
PID	Proportion-integral-derivative
ILC	Iterative learning control
DC	Disturbance compensation
LPIDDC	Iterative learning PID control strategy with disturbance compensation
PM	Permanent magnet
PIDDC	PID with Disturbance compensation
LPID	Iterative learning feed-forwad PID
